# Heart-Rate Recovery at 1 Min After Exercise Predicts Response to Balloon Pulmonary Angioplasty in Patients With Inoperable Chronic Thromboembolic Pulmonary Hypertension

**DOI:** 10.3389/fcvm.2022.795420

**Published:** 2022-02-18

**Authors:** Yi Zhang, Xin Li, Qin Luo, Qing Zhao, Qixian Zeng, Tao Yang, Qi Jin, Lu Yan, Anqi Duan, Xiuping Ma, Chenhong An, Changming Xiong, Zhihui Zhao, Zhihong Liu

**Affiliations:** ^1^Center for Pulmonary Vascular Diseases, National Center for Cardiovascular Diseases, Fuwai Hospital, Chinese Academy of Medical Sciences and Peking Union Medical College, Beijing, China; ^2^Department of Cardiology, Zhongshan Hospital, Fudan University, Shanghai, China

**Keywords:** chronic thromboembolic pulmonary hypertension, balloon pulmonary angioplasty, heart-rate recovery at 1 min, cardiac autonomic function, prognosis

## Abstract

**Background:**

Dysfunction of autonomic nervous system plays an important role in the development of pulmonary hypertension. The present study aimed to investigate the interaction between balloon pulmonary angioplasty (BPA) and cardiac autonomic function by using heart-rate recovery at 1 min (HRR1) after exercise as a surrogate marker.

**Methods and Results:**

We retrospectively enrolled 89 consecutive patients with inoperable chronic thromboembolic pulmonary hypertension who underwent BPA from May, 2018 to Jan, 2021. According to hemodynamics at follow-up, patients were categorized as BPA responders if they met one or both of the following criteria: (1) mean pulmonary arterial pressure ≤ 30 mmHg and (2) a reduction of pulmonary vascular resistance ≥ 30%. Compared with baseline, HRR1 tended to increase within 7 days after the first BPA session, and this improvement persisted at follow-up. HRR1 at baseline and at follow-up were associated with well-validated markers of CTEPH severity, including N-terminal pro-brain natriuretic peptide, mean pulmonary arterial pressure and pulmonary vascular resistance. Furthermore, the change of HRR1 from baseline to follow-up was also associated with the change of those variables. After adjustment for confounders, baseline HRR1 was still a strong independent predictor of BPA outcome. Receiver operator characteristic curve analysis showed that the cutoff value for HRR1 in predicting BPA outcome was 19 beats.

**Conclusions:**

BPA could significantly improve HRR1, suggesting the alleviation of sympathovagal imbalance. Easily available and non-invasive HRR1 seems to be a useful tool in predicting outcome of BPA and dynamically monitoring the efficacy of BPA.

## Introduction

Chronic thromboembolic pulmonary hypertension (CTEPH) is featured by organized chronic thrombi in proximal or distal pulmonary arteries and small-vessel remodeling in non-occluded areas, which increases pulmonary arterial pressure and results in right-sided heart failure (1). The prognosis of CTEPH is poor, with a 5-year survival rate of 10% for patients with a mean pulmonary arterial pressure (mPAP) > 50 mmHg (2).

Currently, pulmonary endarterectomy is the first therapeutic option for CTEPH (3). However, as many as 40% of patients are ineligible for surgical intervention due to reasons like severe comorbidities, distal lesions, and other patient specific factors (4, 5). Over the past decade, refined balloon pulmonary angioplasty (BPA) is emerging as an alternative option for inoperable CTEPH. Increasing evidences suggest that BPA could significantly improve hemodynamics, exercise tolerance and pulmonary function (6, 7).

Sympathetic, parasympathetic, and sensory nerve fibers innervate the pulmonary vasculature (8). In general, sympathetic nerve stimulation causes vasoconstriction of pulmonary vasculature and vagal stimulation results in vasodilation. Dysfunction of autonomic nervous system plays an important role in the development of pulmonary hypertension (8, 9). Moreover, it has been demonstrated that pulmonary artery denervation could improve the hemodynamics and exercise capacity of patients with pulmonary hypertension (10–13).

Heart-rate recovery at 1 min (HRR1) after exercise is a widely recognized surrogate marker of cardiac autonomic function, which is defined as the change in heart rate (HR) from the maximum workload to 60 s after exercise cession (14). HRR1 is a non-invasive and less time-consuming measure, which can be quickly obtained from routine exercise testing [cardiopulmonary exercise test (CPET) or 6-min walk test], compared with other HR responses, such as HR variability measured with Holter electrocardiography. Previous studies have reported that HRR1 was associated with exercise tolerance, hemodynamics and prognosis in patients with pulmonary arterial hypertension (PAH) (group I pulmonary hypertension) (15–17). Additionally, Inagaki et al. also reported that HRR1 was correlated with hemodynamics in CTEPH (group IV pulmonary hypertension) (18). Recently, we reported that HRR1 could independently predict prognosis in patients with CTEPH (19). However, to the best of our knowledge, no one has systematically studied the interaction between BPA and cardiac autonomic function. The main objectives of the present study were to determine whether BPA could alleviate sympathovagal imbalance and whether baseline HRR1 could predict outcome of BPA.

## Materials and Methods

### Study Design and Participants

This retrospective study was conducted in Fuwai Hospital, Chinese Academy of Medical Sciences (Beijing, China). The study protocol was approved by the Ethics Committee of Fuwai Hospital (Approval NO: 2020-1275). Written informed consent was obtained from each patient. We screened all patients with inoperable CTEPH who underwent BPA from May, 2018 to Jan, 2021. The establishment of CTEPH was based on the 2015 European Society of Cardiology/European Respiratory Society guidelines (4). The eligibility for BPA was assessed by a multidiscipline team, consisting of a surgeon specializing in pulmonary endarterectomy, an interventional cardiologist specializing in BPA and a physician specializing in pulmonary hypertension. By design, patients were excluded if they: (1) did not have baseline HRR1 data; (2) did not undergo right heart catheterization (RHC) at follow-up; (3) were using beta-blockers or other antiarrhythmic agents. The following clinical data were collected via an electronic medical record system by two independent reviewers: demographics, World Health Organization functional class (WHO-FC), N-terminal pro-brain natriuretic peptide (NT-proBNP), arterial oxygen saturation (S_a_O_2_), 6-min walk distance (6MWD), targeted therapy at baseline, anticoagulants, parameters derived from echocardiography, CPET and RHC, the number of BPA sessions, the number of dilated subsegmental vessels, and the time interval between baseline and reevaluation RHC. Any discordance was resolved by the supervisors (ZHZ and ZHL).

### RHC and BPA Procedure

In our center, a single catheterization laboratory visit consists of one BPA session and two RHC measurement. The first RHC measurement was performed before the initiation of BPA procedure to acquire the baseline hemodynamics. The second RHC measurement was performed immediately after the BPA procedure to acquire immediate post-operative hemodynamics. The detailed protocols of RHC and BPA have been provided in our previous publications (20, 21). Briefly, RHC was performed to measure hemodynamics, including mixed venous oxygen saturation, right atrial pressure, right ventricular pressure, mPAP, pulmonary arterial wedge pressure (PAWP), cardiac output (calculated by Fick's method) and pulmonary vascular resistance (PVR). After RHC, we performed pulmonary angiography, in anterior-posterior and lateral (60 degree) projections, to acquire overall view of the filling defect. Subsequently, a 70 cm 6F-7F long sheath (Flexor^®^ Check-Flo^®^ Introducer; Cook Medical, Bloomington, IN, USA), via the right femoral vein, was inserted into the lobar pulmonary artery to introduce a 6F guiding catheter (Multi-purpose [Cordis Corporation, Bridgewater, New Jersey, USA] or Amplatz Left [Terumo^®^ Heartrail™ II; Terumo Corporation, Tokyo, Japan] or Judkins Right Tokyo, Japan] or Judkins Right [Terumo^®^ Heartrail™ II; Terumo Corporation, Tokyo, Japan]). Based on selective pulmonary angiography, a 0.014-inch guidewire (Hi-Torque Pilot 50; Abbot, Santa Clara, CA, USA) was passed across the target lesion. To reduce the risk of complications, a 2.0 ×20 mm balloon was used at initial dilation, while smaller balloons may also be used for subtotal or total occlusion lesions. The balloon size was gradually increased in the subsequent BPA sessions according to the reference vessel diameter. Inflation pressure was dynamically adjusted, and selective angiography was performed to confirm vascular filling. Assessment of WHO-FC, NT-proBNP, S_a_O_2_, 6MWD, echocardiography and CPET were performed within 7 days prior to and after each BPA procedure. Follow-up reevaluation, including RHC and CPET, would be performed over 3 months after the last BPA session.

### Cardiopulmonary Exercise Test

The detailed protocols of CPET have been provided in our previous publications (22, 23). Briefly, an incremental symptom-limited exercise test was performed by the same examiner on an upright cycle ergometer using the COSMED Quark CPET system (COSMED, Rome, Italy). Three minutes of rest were followed by 3 min of unloaded pedaling, and progressively increasing workload by 5–30 W/min in a ramp pattern to maximum tolerance. HRR1 was defined as the change in HR from the maximum workload to 60 s after the completion of CPET. Oxygen consumption at peak (VO_2_@Peak) was defined as the highest 30-s average of oxygen consumption in the last minute of exercise. HR at peak represented the highest HR observed during the exercise protocol. HR at recovery was defined as the value of HR at the moment when exercise stopped. ΔHR was defined as (HR at peak—HR at rest). HR acceleration time was defined as the time taken to increase to 75% of ΔHR (3 min of rest was not included). Slope of increased HR was defined as 75% of ΔHR/HR acceleration time (18).

### Definition of BPA Responders and Non-responders

According to the results of reevaluation RHC at follow-up (over 3 months after the last BPA session), patients were categorized as BPA responders or BPA non-responders. In line with previous publications (24), the BPA responders were defined as patients who met one or both of the following criteria: (1) mPAP ≤ 30 mmHg and (2) a reduction of PVR ≥ 30%. Correspondingly, patients with a mPAP > 30 mmHg and a reduction of PVR <30% at follow-up were categorized as BPA non-responders.

### Statistical Analysis

Continuous variables are presented as mean ± standard deviation or median (interquartile range). Categorical variables are given as counts or percentages. Comparison between BPA responders and non-responders were made using an independent-sample *t*-test, the Mann–Whitney *U*-test or the Chi-square test, as appropriate. Two-way analyses of variance were used to compare HRR1 at baseline, after the first BPA session and at follow-up with Tukey's test for multiple comparisons. Correlations between HRR1 and other variables were examined by using Spearman correlation coefficient. The association between baseline HRR1 and BPA outcome was evaluated by using logistic regression model. Univariate logistic regression was firstly performed to identify potential predictors of BPA success. Subsequently, variables with clinical significance or *P* < 0.100 in univariate analysis were selected for multivariable logistic regression (enter method). Receiver operator characteristic (ROC) curve analysis was performed to determine the optimal cutoff of HRR1 in predicting BPA outcome. A two-sided *P* < 0.05 was considered statistically significant. Statistical analysis was performed with SPSS 25.0 (IBM SPSS Corp.; Armonk, NY, USA) and Prism GraphPad 8 (GraphPad Software, LaJolla, CA, USA).

## Results

### Patient Enrollment

One hundred and twenty six patients underwent BPA from May, 2018 to Jan, 2021. Of these patients, 37 were excluded for missing baseline HRR1 data (*n* = 12), no reevaluation RHC at follow-up (*n* = 22) and using beta-blockers or other antiarrhythmic agents (*n* = 3). Among the remaining 89 patients, a total of 206 BPA sessions were performed [2.0 (interquartile range, 1.0-3.0)/per patient], with 1343 subsegmental vessels dilated [14.0 (interquartile range, 7.5–19.0) /per patient]. According to hemodynamics at follow-up, 53 were categorized as BPA responders and 36 patients as BPA non-responders.

### Baseline Characteristics

Baseline characteristics of BPA responders and non-responders are summarized in Table 1. Compared with BPA non-responders, responders had lower levels of NT-proBNP, higher HRR1 and underwent more BPA sessions. Of note, mPAP (50.8 ± 11.9 mmHg vs. 51.5 ± 10.9 mmHg, *P* = 0.861) and PVR (10.2 ± 4.4 wood units vs. 10.0 ± 3.6 wood units, *P* = 0.855) at baseline were comparable between BPA responders and non-responders.

**Table 1:** Baseline characteristics of BPA responders and non-responders.

**Variables**	**All patients (*n =* 89)**	**Responders (*n =* 53)**	**Non-responders (*n =* 36)**	*P*-value^*^
Age, years	58.4 ± 11.6	58.0 ± 11.9	58.9 ± 11.3	0.607
Female, n (%)	47.0 (52.8)	28 (52.8)	19 (52.8)	0.996
Body mass index, kg/m^2^	24.0 ± 3.3	24.1 ± 3.4	23.7 ± 3.3	0.552
WHO FC				0.082
I or II, n (%)	37.0 (41.6)	26 (49.1)	11 (30.6)	
III or IV, n (%)	52.0 (58.4)	27 (50.9)	25 (69.4)	
NT-proBNP, ng/L	814.0 (195.7, 1780.5)	497.0 (107.2, 1450.0)	1052.0 (460.2, 2460)	**0.019**
S_a_O_2_, %	91.6 ± 3.1	91.9 ± 2.7	91.2 ± 3.7	0.380
6MWD, m	366.5 ± 110.5	381.6 ± 96.0	343.9 ± 127.4	0.124
Targeted therapy				0.399
None, n (%)	36 (40.4)	25 (47.2)	11 (30.6)	
Monotherapy
ERAs, n (%)	6 (6.7)	3 (5.7)	3 (8.3)	
PDE-5is, n (%)	21 (23.6)	9 (17.0)	12 (33.3)	
sGCs, n (%)	16 (18.0)	8 (15.1)	8 (22.2)	
Combination
ERAs+PDE-5is, n (%)	9 (10.1)	7 (13.2)	2 (5.6)	
ERAs+ sGCs, n (%)	1 (1.1)	1 (1.9)	0	
Anticoagulants				0.414
Warfarin, n (%)	57 (64.0)	31 (58.5)	26 (72.2)	
Rivaroxaban, n (%)	29 (32.6)	20 (37.7)	9 (25.0)	
Dabigatran, n (%)	3 (3.4)	2 (3.8)	1 (2.8)	
Echocardiography
LVED, mm	41.0 ± 5.4	40.9 ± 5.8	41.0 ± 4.9	0.621
RVED, mm	32.3 ± 6.2	31.7 ± 5.9	33.1 ± 6.7	0.451
RVED/LVED	0.8 ± 0.2	0.8 ± 0.2	0.8 ± 0.2	0.841
LVEF, %	65.0 ± 5.5	65.1 ± 5.8	64.9 ± 5.0	0.923
TRV, m/s	4.3 ± 0.6	4.3 ± 0.7	4.4 ± 0.6	0.543
Cardiopulmonary exercise test
HR at rest, beats/min	77.7 ± 13.1	77.2 ± 12.2	78.5 ± 14.4	0.645
HR at peak, beats/min	124.3 ± 20.7	127.0 ± 19.1	120.4 ± 22.5	0.140
HR at recovery^a^, beats/min	122.8 ± 24.4	126.6 ± 19.6	117.2 ± 29.5	0.076
HRR1, beats	16.0 (10.0, 22.5)	17.0 (11.0, 26.5)	13.0 (8.0, 17.0)	**0.018**
ΔHR^b^, beats	46.1 ± 20.1	49.8 ± 20.5	41.9 ± 18.7	0.067
HR acceleration time^c^, s	407.9 ± 135.6	429.6 ± 111.5	376.1 ± 161.1	0.068
Slope of increased HR^d^	0.09 (0.07, 0.11)	0.09 (0.07, 0.11)	0.08 (0.07, 0.11)	0.701
VO_2_@Peak, mL/min/kg	12.5 ± 3.5	12.8 ± 3.9	12.0 ± 2.6	0.278
VE/VCO_2_ slope	49.2 ± 9.5	49.1 ± 9.7	49.3 ± 9.2	0.969
Hemodynamics
S_v_O_2_, %	69.2 ± 5.2	69.9 ± 5.1	68.2 ± 5.3	0.137
mRAP, mmHg	8.0 (6.0, 9.0)	7.8 ± 3.1	8.2 ± 3.8	0.708
mPAP, mmHg	51.1 ± 11.4	50.8 ± 11.9	51.5 ± 10.9	0.861
PAWP, mmHg	10.0 ± 3.2	9.3 ± 3.1	10.8 ± 3.3	0.050
Cardiac index, L/min/m^2^	3.0 ± 0.7	3.0 ± 0.7	3.0 ± 0.7	0.635
PVR, wood units	10.1 ± 4.1	10.2 ± 4.4	10.0 ± 3.6	0.855
BPA procedure
Number of BPA sessions	2.0 (1.0, 3.0)	2.0 (1.0, 4.0)	2.0 (1.0, 3.0)	**0.039**
Number of dilated subsegmental vessels	14.0 (7.5, 19.0)	14.0 (9.0, 22.5)	12.0 (7.0, 17.0)	0.115
Time interval*^$^*, days	227.0 (117.0, 422.0)	287.0 (161.0, 486.5)	203.5 (100.8, 372.0)	0.122

*Data are presented as mean ± standard deviation, median (interquartile range) or number (percentage). BPA, balloon pulmonary angioplasty; ERAs, Endothelin receptor antagonists; HR, heart rate; HRR1, heart-rate recovery at 1 min; LVED, Left ventricular end-diastolic diameter; LVEF, Left ventricular ejection fraction; mPAP, Mean pulmonary arterial pressure; mRAP, Mean right atrial pressure; NT-proBNP, N-terminal pro-brain natriuretic peptide; PAWP, Pulmonary arterial wedge pressure; PDE-5is, Phosphodiesterase-5 inhibitors; PVR, Pulmonary vascular resistance; RVED, Right ventricular end-diastolic diameter; 6MWD, 6-min walk distance; sGCs, Soluble guanylate cyclase stimulators; S_a_O_2_, arterial oxygen saturation; S_V_O_2_, Mixed venous oxygen saturation; TRV, tricuspid regurgitation velocity; VE/VCO_2_ slope, Minute ventilation/carbon dioxide output slope; VO_2_@Peak, Peak oxygen consumption; WHO FC, World Health Organization functional class*.

$ *Time interval between baseline and follow-up*.

* *Responders vs. non-responders*.

a *The value of HR at the moment when exercise stopped*.

b *HR at peak minus HR at rest*.

c *The time taken to increase to 75% of ΔHR (3 min of rest was not included)*.

d *75% of ΔHR/HR acceleration time. Bold values mean P < 0.05*.

We also compared baseline characteristics of the included and excluded patients. Both groups were comparable in terms of demographics, exercise capacity, cardiac function and morphology, hemodynamics, and targeted therapy at baseline except higher proportion of WHO FC I/II in the included patients (Supplementary Table 1).

### Clinical Assessments at Follow-Up

The clinical status of BPA responders and non-responders at follow-up is presented in Table 2. Like baseline, BPA responders still had higher proportion of WHO FC I/II, lower levels of NT-proBNP, and higher HRR1 at follow-up. More importantly, BPA responders had higher S_a_O_2_, more favorable echocardiographic parameters [reflected by smaller right ventricular end-diastolic diameter/left ventricular end-diastolic diameter (RVED/LVED), greater left ventricular ejection fraction and lower tricuspid regurgitation velocity], lower VE/VCO_2_ slope and better hemodynamics (reflected by higher mixed venous oxygen saturation, lower mPAP, higher cardiac index and lower PVR) than BPA non-responders, even though these variables were comparable at baseline between the two groups. Among BPA responders, 21 achieved a mPAP ≤ 30 mmHg and 4 achieved a mPAP < 25 mmHg.

**Table 2:** Re-assessment of BPA responders and non-responders at follow-up^*^.

**Variables**	**Responders (*n =* 53)**	**Non-responders (*n =* 36)**	***P*-value**
WHO FC			**0.023**
I or II, n (%)	48.0 (90.6)	26.0 (72.2)	
III or IV, n (%)	5.0 (9.4)	10.0 (27.8)	
NT-proBNP, ng/L	103.0 (56.9, 237.7)	356.0 (158.2, 800.9)	**0.001**
S_a_O_2_, %	93.7 ± 2.6	91.3 ± 6.2	**0.007**
6MWD, m	436.8 ± 86.2	417.3 ± 91.3	0.396
Echocardiography
LVED, mm	44.4 ± 4.6	42.6 ± 4.0	0.097
RVED, mm	28.1 ± 4.9	30.2 ± 6.4	0.089
RVED/LVED	0.6 ± 0.1	0.7 ± 0.2	**0.027**
LVEF, %	66.4 ± 4.8	62.9 ± 5.7	**0.008**
TRV, m/s	3.6 ± 0.7	4.2 ± 0.6	**<0.001**
Cardiopulmonary exercise test
HR at rest, beats/min	74.7 ± 13.3	75.9 ± 11.5	0.561
HR at peak, beats/min	126.9 ± 18.5	121.2 ± 21.0	0.155
HR at recovery^a^, beats/min	123.6 ± 25.3	120.3 ± 20.8	0.247
HRR1, beats	24 (17.5, 32)	20 (13.0, 28.0)	**0.048**
ΔHR^b^, beats	52.2 ± 17.1	45.3 ± 18.3	0.075
HR acceleration time^c^, s	451.3 ± 53.8	419.3 ± 115.0	0.446
Slope of increased HR^d^	0.09 (0.07, 0.10)	0.08 (0.06, 0.10)	0.444
VO_2_@Peak, mL/min/kg	15.0 ± 3.8	13.5 ± 3.6	0.081
VE/VCO_2_ slope	41.3 ± 7.9	45.3 ± 7.6	**0.025**
Hemodynamics
S_v_O_2_, %	72.6 ± 4.7	69.6 ± 5.7	**0.007**
mRAP, mmHg	6.5 ± 3.0	7.0 ± 3.3	0.536
mPAP, mmHg	34.9 ± 9.2	46.2 ± 10.5	**<0.001**
PAWP, mmHg	10.3 ± 3.4	9.9 ± 3.6	0.567
Cardiac index, L/min/m^2^	3.5 ± 0.9	3.1 ± 1.0	**0.020**
PVR, wood units	5.1 ± 2.3	9.1 ± 3.4	**<0.001**
Decrease of mPAP, %	−31.8 (−39.4, −19.3)	−10.8 (−18.8, −16.3)	**<0.001**
Decrease of PVR, %	−45.3 (−62.0, −35.5)	−12.2 (−25.1, 0.1)	**<0.001**

*Data are presented as mean ± standard deviation, median (interquartile range) or number (percentage). BPA, balloon pulmonary angioplasty; HR, heart rate; HRR1, heart-rate recovery at 1 min; LVED, Left ventricular end-diastolic diameter; LVEF, Left ventricular ejection fraction; mPAP, Mean pulmonary arterial pressure; mRAP, Mean right atrial pressure; NT-proBNP, N-terminal pro-brain natriuretic peptide; PAWP, Pulmonary arterial wedge pressure; PVR, Pulmonary vascular resistance; RVED, Right ventricular end-diastolic diameter; 6MWD, 6-min walk distance; S_a_O_2_, arterial oxygen saturation; S_V_O_2_, Mixed venous oxygen saturation; TRV, tricuspid regurgitation velocity; VE/VCO_2_ slope, Minute ventilation/carbon dioxide output slope; VO_2_@Peak, Peak oxygen consumption; WHO FC, World Health Organization functional class*.

* *Over 3 months after the last BPA session*.

a *The value of HR at the moment when exercise stopped*.

b *HR at peak minus HR at rest*.

c *The time taken to increase to 75% of ΔHR (3 min of rest was not included)*.

d *75% of ΔHR/HR acceleration time. Bold values mean P < 0.05*.

### The Effect of BPA on HRR1

**Figure 1:**
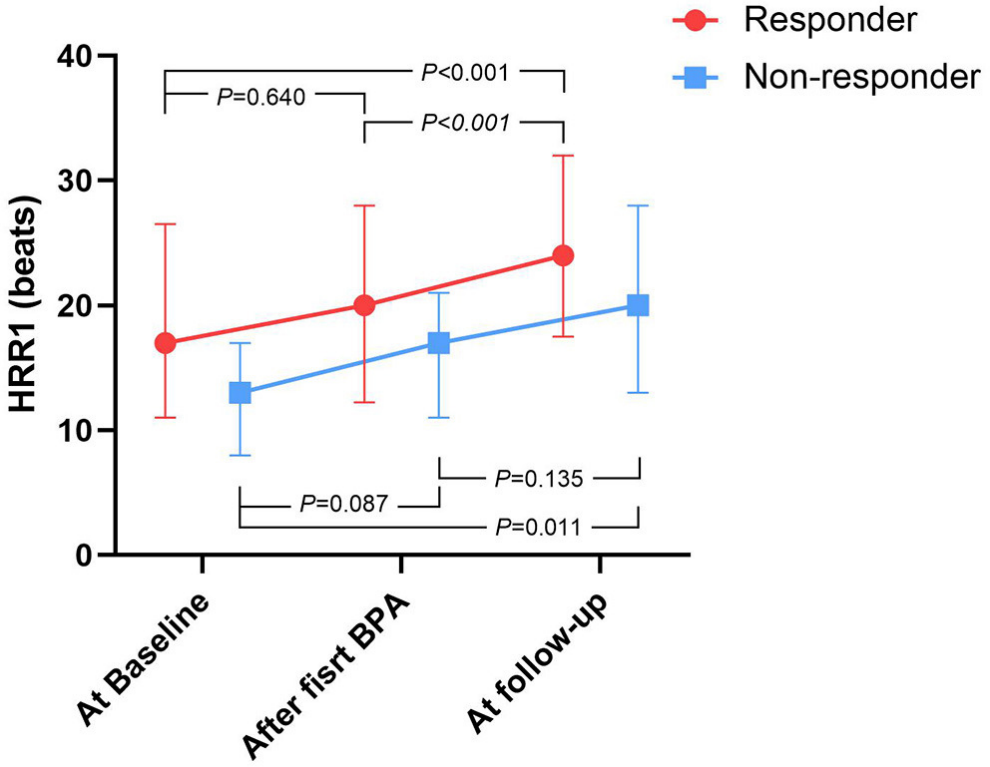
The time course of HRR1 values during BPA procedure, stratified by BPA responders and non-responders. BPA, Balloon pulmonary angioplasty; HRR1, heart-rate recovery at 1 min. At baseline: within 7 days prior to the first BPA session, after first BPA: within 7 days after the first BPA session, At follow-up: over 3 months after the last BPA session.

Figure 1 shows that, compared with baseline, HRR1 tended to increase within 7 days after the first BPA session in both BPA responders and non-responders, and this improvement persisted at follow-up.

### Correlation Between HRR1 and Well-Validated Markers of CTEPH Severity

As shown in Table 3, HRR1 at baseline and follow-up were associated with NT-proBNP, VO_2_@Peak, VE/VCO_2_ slope, RVED/LVED, mPAP, and PVR. Furthermore, the absolute change of HRR1 from baseline to follow-up was associated with the absolute change of NT-proBNP, VO_2_@Peak, VE/VCO_2_ slope and RVED/LVED from baseline to follow-up. Additionally, the absolute change of HRR1 from baseline to follow-up was also associated with the number of BPA sessions, the number of BPA dilated subsegmental vessels, and the time interval between baseline and follow-up.

**Table 3:** Correlation between HRR1 and well-validated markers of CTEPH severity.

**Variable**	**HRR1 at baseline**	**HRR1 at follow-up**	**ΔHRR1**
NT-proBNP at baseline	*r =*−0.422, ***P** **<*** **0.001**		
NT-proBNP at follow-up		*r =*−0.302, ***P** **=*** **0.006**	
ΔNT-proBNP			*r =*−0.225, ***P** **=*** **0.049**
S_a_O_2_ at baseline	*r =* 0.248, ***P** **=*** **0.019**		
S_a_O_2_ at follow-up		*r =* 0.172, *P =* 0.126	
ΔS_a_O_2_			*r =* 0.037, *P =* 0.750
VO_2_@Peak at baseline	*r =* 0.540, ***P** **<*** **0.001**		
VO_2_@Peak at follow-up		*r =* 0.410, ***P** **<*** **0.001**	
ΔVO_2_@Peak			*r =* 0.293, ***P** **=*** **0.013**
VE/VCO_2_ slope at baseline	*r =*−0.548, ***P** **<*** **0.001**		
VE/VCO_2_ slope at follow-up		*r =*−0.481, ***P** **<*** **0.001**	
ΔVE/VCO_2_ slope			*r =*−0.333, ***P** **=*** **0.004**
RVED/LVED at baseline	*r =*−0.250, ***P** **=*** **0.018**		
RVED/LVED at follow-up		*r =*−0.261, ***P** **=*** **0.018**	
ΔRVED/LVED			*r =*−0.089, *P =* 0.439
mPAP at baseline	*r =*−0.332, ***P** **=*** **0.001**		
mPAP at follow-up		*r =*−0.297, ***P** **=*** **0.007**	
ΔmPAP			*r =*−0.134, *P =* 0.245
PVR at baseline	*r =*−0.412, ***P** **<*** **0.001**		
PVR at follow-up		*r =*−0.311, ***P** **=*** **0.005**	
ΔPVR			*r =*−0.069, *P =* 0.557
Number of BPA sessions			*r =* 0.411, ***P** **<*** **0.001**
Number of dilated subsegmental vessels			*r =* 0.445, ***P** **<*** **0.001**
Time interval*			*r =* 0.298, ***P** **=*** **0.008**

BPA, balloon pulmonary angioplasty; CTEPH, Chronic thromboembolic pulmonary hypertension; HRR1, heart-rate recovery at 1 min; LVED, Left ventricular end-diastolic diameter; LVEF, Left ventricular ejection fraction; mPAP, Mean pulmonary arterial pressure; NT-proBNP, N-terminal pro-brain natriuretic peptide; PVR, Pulmonary vascular resistance; RVED, Right ventricular end-diastolic diameter; S_a_O_2_, arterial oxygen saturation; VE/VCO_2_ slope, Minute ventilation/ carbon dioxide output slope; VO_2_@Peak, Peak oxygen consumption. *Time interval between baseline and follow-up. Δ means the absolute change of these variables from baseline to follow-up. Bold values mean P < 0.05.

### Predictors of BPA Responders

In univariate logistic regression, WHO FC, NT-proBNP, HR at recovery, HRR1, ΔHR, HR acceleration time, PAWP, the number of BPA sessions, the number of dilated subsegmental vessels, and the time interval from baseline to follow-up had a *P* < 0.100 (Table 4). Age, mPAP and PVR were also included in multivariable logistic regression (enter method) for their clinical significance. The number of dilated subsegmental vessels (*r* = 0.861, *P* < 0.001) and the time interval from baseline to follow-up (*r* = 0.745, *P* < 0.001) were excluded from multivariable logistic regression for their collinearity with the number of BPA sessions. Variables reflecting HR response to exercise (i.e., HR at recovery, HRR1, ΔHR and HR acceleration time) did not enter multivariable logistic regression simultaneously after consideration of sample size and collinearity. Finally, three to seven independent variables were included in multivariable logistic regression based on the number of events observed (25, 26). In model 1, HRR1 was adjusted for the number of dilated subsegmental vessels and PAWP. In subsequent models, HRR1 was further adjusted for the variables in model 1 plus age, NT-proBNP, WHO FC, mPAP, and PVR. In all these 6 multivariable logistic models, HRR1 remained as an independent predictor of BPA responder (Table 5). Similar analysis procedure was also performed for HR at recovery, ΔHR and HR acceleration time. However, no significant association was observed between these variables and BPA outcome (Supplementary Table 2).

**Table 4:** Univariate logistic regression analysis for BPA responders.

**Variable**	**OR**	**95% CI**	***P*-value**
Age	0.993	0.957–1.031	0.712
Female	1.002	0.429–2.340	0.996
Body mass index	1.040	0.914–1.184	0.547
WHO FC	0.457	0.188–1.113	0.085
NT-proBNP	1.000	0.999–1.000	0.074
S_a_O_2_	1.074	0.936–1.232	0.308
6MWD	1.003	0.999–1.007	0.128
RVED/LVED	0.476	0.066–3.450	0.463
LVEF	1.005	0.929–1.086	0.905
TRV	0.810	0.413–1.587	0.539
HR at rest	0.992	0.960–1.025	0.641
HR at peak	1.016	0.995–1.038	0.142
HR at recovery^a^	1.017	0.997–1.037	0.088
HRR1	1.041	0.997–1.088	0.071
ΔHR^b^	1.021	0.998–1.044	0.071
HR acceleration time^c^	1.003	1.000–1.006	0.081
Slope of increased HR^d^	0.035	0.000–15.984	0.284
VO_2_@Peak	1.075	0.943–1.226	0.277
VE/VCO_2_ slope	0.998	0.953–1.045	0.931
S_v_O_2_	1.065	0.980–1.158	0.138
mRAP	0.964	0.851–1.092	0.566
mPAP	0.994	0.958–1.032	0.762
PAWP	0.857	0.741–0.990	**0.037**
Cardiac Index	1.106	0.600–2.038	0.747
PVR	1.014	0.911–1.127	0.802
Number of BPA sessions	1.545	1.067–2.236	**0.021**
Number of dilated subsegmental vessels	1.062	1.004–1.123	**0.037**
Time interval^*^	1.002	1.000–1.004	0.080

*BPA, balloon pulmonary angioplasty; HRR1, heart-rate recovery at 1 min; LVED, Left ventricular end-diastolic diameter; LVEF, Left ventricular ejection fraction; mPAP, Mean pulmonary arterial pressure; mRAP, Mean right atrial pressure; NT-proBNP, N-terminal pro-brain natriuretic peptide; PAWP, Pulmonary arterial wedge pressure; PVR, Pulmonary vascular resistance; RVED, Right ventricular end-diastolic diameter; 6MWD, 6-min walk distance; S_V_O_2_, Mixed venous oxygen saturation; TRV, tricuspid regurgitation velocity; VE/VCO_2_ slope, Minute ventilation/ carbon dioxide output slope; VO_2_@Peak, Peak oxygen consumption; WHO FC, World Health Organization functional class*.

* *Time interval between baseline and follow-up*.

a *The value of HR at the moment when exercise stopped*.

b *HR at peak minus HR at rest*.

c *The time taken to increase to 75% of ΔHR (3 min of rest was not included)*.

d *75% of ΔHR/HR acceleration time. Bold values mean P < 0.05*.

**Table 5:** Multivariable logistic regression analysis for BPA responders.

**Model**	**Variable**	**OR**	**95% CI**	***P*-value**
1	HRR1	1.059	1.010–1.110	**0.017**
	Number of BPA sessions	1.802	1.173–2.771	**0.007**
	PAWP	0.824	0.700–0.971	**0.021**
2	HRR1	1.066	1.013–1.121	**0.013**
	Number of BPA sessions	1.881	1.199–2.950	**0.006**
	PAWP	0.826	0.700–0.975	**0.024**
	Age	1.018	0.972–1.065	0.453
3	HRR1	1.056	1.002–1.113	**0.041**
	Number of BPA sessions	1.896	1.198–3.002	**0.006**
	PAWP	0.816	0.689–0.968	**0.019**
	Age	1.017	0.971–1.066	0.463
	NT-proBNP	1.000	0.999–1.000	0.239
4	HRR1	1.057	1.001–1.116	**0.048**
	Number of BPA sessions	1.898	1.197–3.010	**0.006**
	PAWP	0.816	0.689–0.968	**0.019**
	Age	1.017	0.971–1.066	0.464
	NT-proBNP	1.000	0.999–1.000	0.270
	WHO FC	1.042	0.309–2.505	0.948
5	HRR1	1.061	1.004–1.122	**0.037**
	Number of BPA sessions	1.860	1.167–2.966	**0.009**
	PAWP	0.805	0.676–0.959	**0.015**
	Age	1.021	0.973–1.071	0.394
	NT-proBNP	1.000	0.999–1.000	0.216
	WHO FC	0.943	0.273–3.258	0.927
	mPAP	1.021	0.972–1.071	0.410
6	HRR1	1.062	1.004–1.123	**0.034**
	Number of BPA sessions	1.894	1.177–3.050	**0.009**
	PAWP	0.826	0.695–0.982	**0.031**
	Age	1.018	0.971–1.068	0.458
	NT-proBNP	1.000	0.999–1.000	0.126
	WHO FC	0.803	0.223–2.887	0.737
	PVR	1.121	0.952–1.319	0.169

*BPA, balloon pulmonary angioplasty; HRR1, heart-rate recovery at 1 min; mPAP, Mean pulmonary arterial pressure; NT-proBNP, N-terminal pro-brain natriuretic peptide; PAWP, Pulmonary arterial wedge pressure; PVR, Pulmonary vascular resistance; WHO FC, World Health Organization functional class. Bold values mean P < 0.05*.

**Figure 2:**
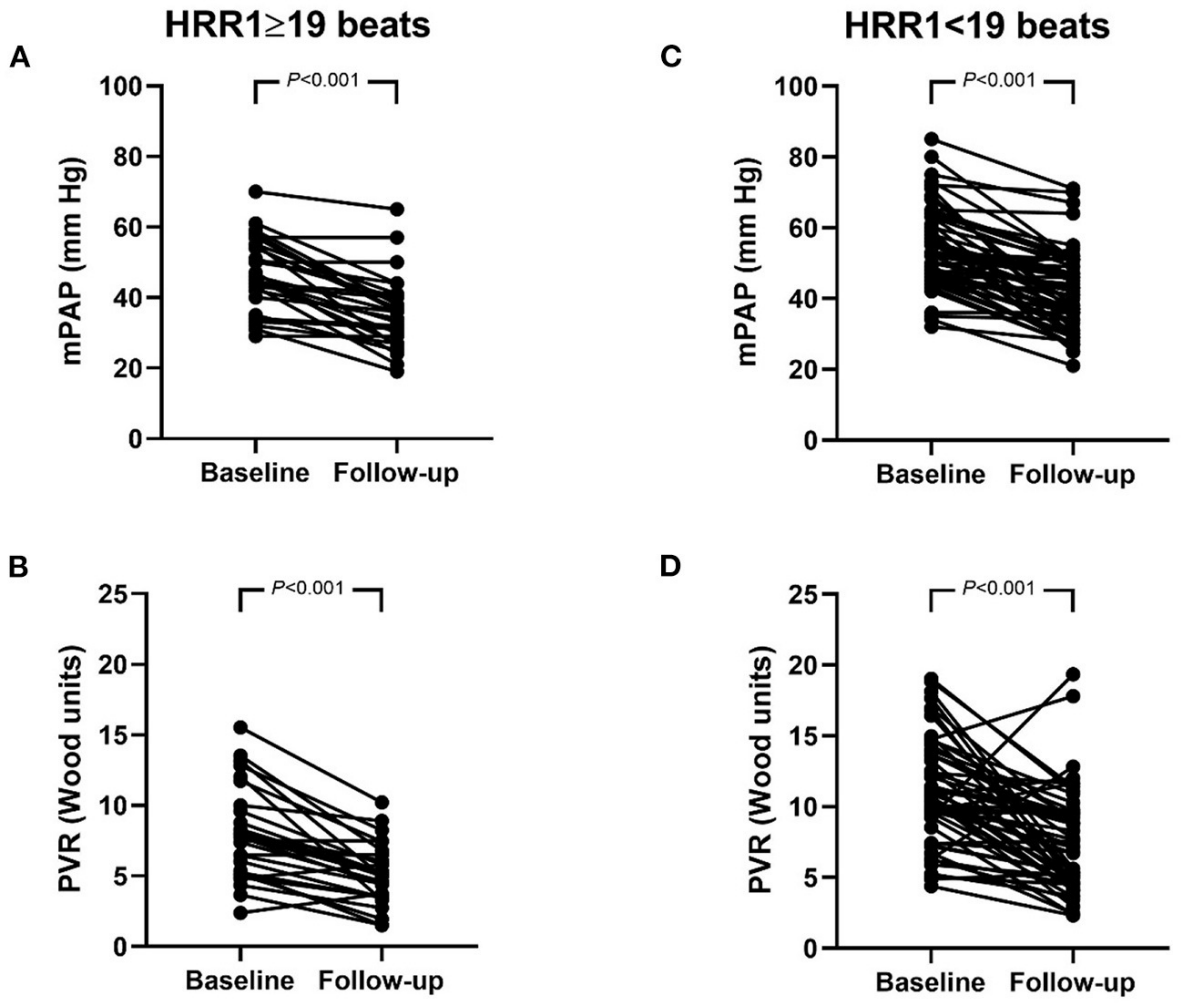
**(A–D)** Hemodynamics at baseline and follow-up in patients with HRR1 ≥ 19 beats and < 19 beats. mPAP **(A)** and PVR **(B)** in patients with HRR1 ≥ 19 beats. mPAP **(C)** and PVR **(D)** in patients with HRR1 < 19 beats. At baseline, HRR1 ≥ 19 beats vs. HRR1 < 19 beats (mPAP: 46.2 ± 10.2 mm Hg vs. 53.5 ± 11.3 mmHg, *P* = 0.004; PVR: 8.0 ± 3.2 wood units vs. 11.3 ± 4.0 wood units, *P* < 0.001). At follow-up, HRR1 ≥ 19 beats vs. HRR1 < 19 beats (mPAP: 35.5 ± 10.0 mm Hg vs. 41.4 ± 11.3 mmHg, *P* = 0.012; PVR: 5.1 ± 2.1 wood units vs. 7.4 ± 3.6 wood units, *P* = 0.003). HRR1, heart-rate recovery at 1 min; mPAP, mean pulmonary arterial pressure; PVR, pulmonary vascular resistance.

Using ROC curve analysis, with the largest sum of sensitivity and specificity chosen, the cutoff value for HRR1 in predicting BPA responders was 19 beats, with an area under the curve of 0.643 (95% CI: 0.528–0.758). According to this cutoff value, 30 patients were classified into HRR1 ≥ 19 beats and 59 patients into HRR1 < 19 beats. The number of BPA sessions [median (interquartile range): 2.0 (1.0, 3.0) vs. 2.0 (1.0, 3.0), *P* = 0.561], the number of dilated subsegmental vessels [median (interquartile range): 10.5 (7.0, 17.0) vs. 14.0 (9.0, 19.0), *P* = 0.101], and the time interval between baseline and reevaluation RHC [median (interquartile range): 267.5 (115.3, 425.3) days vs. 210.0 (115.0, 427.0) days, *P* = 0.801] were comparable between patients with HRR1 ≥ 19 beats and < 19 beats. Compared with baseline, mPAP and PVR improved at follow-up in both HRR1 ≥ 19 beats and < 19 beats groups (Figure 2). However, the proportion of BPA responders was significantly higher in patients with HRR1 ≥ 19 beats (80.0% vs. 49.1%, *P* = 0.005).

## Discussion

The most important finding of our study is that easily available and non-invasive HRR1 is a strong predictor of BPA outcome. Second, we found that HRR1 tended to increase within 7 days after the first BPA session, and this improvement persisted at follow-up, suggesting that BPA could alleviate sympathovagal imbalance. Last, our results showed that improvement in HRR1 was associated with improvement in the well-validated markers of CTEPH severity, indicating that HRR1 might serve as a biomarker which could monitor the efficacy of BPA sessions.

### BPA Alleviated Sympathovagal Imbalance

As shown in Figure 1, HRR1 tended to increase within 7 days after the first BPA session and this improvement persisted at follow-up in both BPA responders and non-responders. Similarly, Huo et al. found that the administration of Ambrisentan continuously improved HRR1 in patients with PAH (27). We also found that HRR1 was associated with well-validated markers of CTEPH severity both at baseline and follow-up. More importantly, the change of HRR1 from baseline to follow-up was also correlated with the change of those markers. Therefore, easily available and non-invasive HRR1 might serve as a biomarker which could dynamically monitor the efficacy of BPA sessions.

In healthy subjects, HR is regulated by a sympathovagal balance (28, 29). During exercise, sympathetic activation and parasympathetic withdrawal both contribute to the increase of HR; after peak exercise, sympathetic withdrawal and parasympathetic reactivation both contribute to the recovery of HR (14, 30). Pulmonary hypertension results in right-sided heart failure, which is a syndrome affecting many organs rather than a condition of pure hemodynamic failure. Previous studies have reported sympathetic hyperactivity and parasympathetic hypoactivity in PAH (9, 31), which is considered as an adaptive mechanism for reduced cardiac output (8). The aforementioned mechanism could also be operational in CTEPH. Thus, it is possible that the impaired HRR1 observed in our study reflected potentially continued sympathetic activation and a lack of normal parasympathetic reactivation after peak exercise. We hypothesized that BPA ameliorated hemodynamics, improved right and left ventricular function, increased cardiac output and then alleviated sympathovagal imbalance (reflected by increased HRR1).

### Baseline HRR1 Predicts the Outcome of BPA

To date, there is no widely recognized tools for predicting the efficacy of BPA prior to intervention. In the present study, we found that baseline HRR1 was a strong predictor of BPA outcome. Previous studies have demonstrated that both sympathetic hyperactivity and parasympathetic hypoactivity are associated with pulmonary vascular remodeling and right ventricular dysfunction (8, 9, 32). At baseline, BPA non-responders had lower HRR1 than responders, even though hemodynamics were comparable between both groups. At follow-up, BPA non-responders had reasonably worse hemodynamics and still had lower HRR1 than responders. This indicated that, both at baseline and follow-up, BPA non-responders had more severe sympathovagal imbalance than responders. We speculated that severe sympathovagal imbalance at baseline (reflected by HRR1 < 19 beats) may increase the tone of pulmonary vasculature to a higher level, which weakens the efficacy of the future BPA sessions. Thus, for BPA non-responders with HRR1 < 19 beats at baseline, transcatheter pulmonary artery denervation might serve as a complementary therapy. Romanov et al. reported that, in patients with residual CTEPH after pulmonary endarterectomy, those underwent transcatheter pulmonary artery denervation had greater improvement in hemodynamics and exercise capacity than those treated with riociguat (13).

It should be stressed that we do not mean to imply that HRR1 < 19 beats is a contraindication for BPA. As shown in Figure 2, mPAP and PVR were also decreased significantly after BPA in patients with HRR1 < 19 beats. Our results should be interpreted as: when undergoing similar amount of BPA sessions and dilating similar amount of subsegmental vessels, the percentage of BPA responders were higher in patients with HRR1 ≥ 19 beats at baseline than that in those with HRR1 < 19 beats at baseline (80% vs. 49.1%, *P* = 0.005). Another issue is that the number of BPA sessions is relatively small for both BPA-responders and non-responders in the present study. Non-responders may turn into responders in the future BPA sessions. However, this does not undermine the clinical importance of our results. Because clinicians could anticipate that patients with HRR1 ≥ 19 beats at baseline may achieve a more favorable hemodynamic amelioration with less BPA session and lower medical costs than those with HRR1 < 19 beats.

## Limitations

The main limitation of the study is the inherent biases of a retrospective study. Thirty-seven patients were excluded from the study. Nevertheless, we found that the baseline characteristics were comparable between included and excluded patients. Another limitation is that the autonomic function was indirectly assessed by using HRR1 as a surrogate marker in the present study. The interaction between BPA and the autonomic function needs to be further investigated by using gold standard methods such as the measurement of muscle sympathetic nerve activity.

## Conclusion

BPA could significantly improve HRR1, which indicates the alleviation of sympathovagal imbalance. The change in HRR1 after BPA is associated with the change of well-validated markers of CTEPH severity, which suggests that HRR1 might serve as a biomarker for dynamically monitoring the efficacy of BPA sessions. Baseline HRR1 is a strong independent predictor of BPA outcome.

## Data Availability Statement

The original contributions presented in the study are included in the article/Supplementary Material, further inquiries can be directed to the corresponding author.

## Ethics Statement

The studies involving human participants were reviewed and approved by the Ethics Committee of Fuwai Hospital (Approval No. 2020-1275). The patients/participants provided their written informed consent to participate in this study.

## Author Contributions

ZL and ZZ contributed to the conception of the study and are guarantors of the paper, taking responsibility for the integrity of the work as a whole, from inception to published article. YZ and XL wrote the manuscript. QZh, QZe, TY, QJ, LY, AD, XM, and CA contributed to data collection. ZL, CX, and QL contributed to the acquisition of funding. All authors critically reviewed the manuscript for intellectual content and had final responsibility for the decision to submit for publication, contributed to data analysis, and interpretation.

## Funding

This research article was supported by Beijing Municipal Science and Technology Project [Z181100001718200]; Beijing Municipal Natural Science Foundation [7202168]; CAMS Innovation Fund for Medical Sciences (CIFMS) [2020-I2M-C&T-B-055 and 2021-I2M-C&T-B-032]; Double First-Class Discipline Construction Fund of Peking Union Medical College and Chinese Academy of Medical Sciences [2019E-XK04-02]; the Capital's Funds for Health Improvement and Research (CFH) [2020-2-4033 and 2020-4-4035]; and the Youth Fund of Zhongshan Hospital, Fudan University [Grant No. 2021-016].

## Conflict of Interest

The authors declare that the research was conducted in the absence of any commercial or financial relationships that could be construed as a potential conflict of interest.

## Publisher's Note

All claims expressed in this article are solely those of the authors and do not necessarily represent those of their affiliated organizations, or those of the publisher, the editors and the reviewers. Any product that may be evaluated in this article, or claim that may be made by its manufacturer, is not guaranteed or endorsed by the publisher.
