# Supplementary material for: Heart-Rate Recovery at 1 Min After Exercise Predicts Response to Balloon Pulmonary Angioplasty in Patients With Inoperable Chronic Thromboembolic Pulmonary Hypertension
Source: Front Cardiovasc Med. 2022 Feb 18;9:795420. doi: 10.3389/fcvm.2022.795420 (PMC8894584; doi:10.3389/fcvm.2022.795420)
Supplement: Supplementary file 1 [file Data_Sheet_1.docx]

Table S1 Baseline characteristics of included and excluded patients.

| Variables | Patients included (n=89) | Patients excluded (n=37) | *P* value |
| --- | --- | --- | --- |
| Age, years | 58.4±11.6 | 62.2±8.4 | 0.094 |
| Female, n (%) | 47.0(52.8) | 21.0(56.8) | 0.686 |
| Body mass index, kg/m^2^ | 24.0±3.3 | 22.6±5.0 | 0.163 |
| WHO FC |  |  | **0.015** |
| I or II, n (%) | 37.0(41.6) | 7(18.9) |  |
| III or IV, n (%) | 52.0(58.4) | 30(81.1) |  |
| NT-proBNP, ng/L | 814.0(195.7, 1780.5) | 913.1(199.7, 2383.0) | 0.491 |
| S_a_O_2_, % | 91.6±3.1 | 91.4±2.8 | 0.603 |
| 6MWD, m | 366.5±110.5 | 372.3±109.5 | 1.000 |
| Targeted therapy at baseline |  |  | 0.569 |
| None, n (%) | 36.0(40.4) | 17.0(45.9) |  |
| Mono-therapy/Combination, n (%) | 53.0(59.6) | 20.0(54.1) |  |
| Echocardiography |  |  |  |
| LVED, mm | 41.0±5.4 | 41.0±6.4 | 0.782 |
| RVED, mm | 32.3±6.2 | 33.9±6.6 | 0.146 |
| RVED/LVED | 0.8±0.2 | 0.9±0.3 | 0.439 |
| LVEF, % | 65.0±5.5 | 66.9±6.7 | 0.085 |
| TRV, m/s | 4.3±0.6 | 4.3±0.6 | 0.930 |
| Cardiopulmonary exercise test |  |  |  |
| VO_2_@Peak, mL/min/kg | 12.5±3.5 | 12.3±3.9 | 0.831 |
| VE/VCO_2_ slope | 49.2±9.5 | 45.5±9.4 | 0.119 |
| Hemodynamics |  |  |  |
| S_v_O_2_, % | 69.2±5.2 | 68.3±8.2 | 1.000 |
| mRAP, mmHg | 8.0(6.0, 9.0) | 8.0(6.0, 10.0) | 0.548 |
| mPAP, mmHg | 51.1±11.4 | 48.4±12.2 | 0.243 |
| PAWP, mmHg | 10.0±3.2 | 10.5±3.3 | 0.372 |
| Cardiac index, L/min/m^2^ | 3.0±0.7 | 2.8±0.8 | 0.201 |
| PVR, wood units | 10.1±4.1 | 10.5±5.7 | 0.935 |

Data are presented as mean ± standard deviation, median (interquartile range) or number (percentage). BPA, balloon pulmonary angioplasty; HRR1, heart-rate recovery at one minute; LVED, Left ventricular end-diastolic diameter; LVEF, Left ventricular ejection fraction; mPAP, Mean pulmonary arterial pressure; mRAP, Mean right atrial pressure; NT-proBNP, N-terminal pro-brain natriuretic peptide; PAWP, Pulmonary arterial wedge pressure; PVR, Pulmonary vascular resistance; RVED, Right ventricular end-diastolic diameter; 6MWD, 6-min walk distance; S_a_O_2_, arterial oxygen saturation; S_V_O_2_, Mixed venous oxygen saturation; TRV, tricuspid regurgitation velocity; VE/VCO_2_ slope, Minute ventilation/ carbon dioxide output slope; VO_2_@Peak, Peak oxygen consumption; WHO FC, World Health Organization functional class.

Table S2 Multivariate logistic regression analysis for BPA responders.

| Model | Variable | OR | 95%CI | *P*-value |
| --- | --- | --- | --- | --- |
| 1 | HR at recovery ^a^ | 1.013 | 0.993-1.033 | 0.216 |
|  | Number of BPA sessions | 1.546 | 1.025-2.332 | **0.038** |
|  | PAWP | 0.860 | 0.738-1.001 | 0.052 |
| 2 | HR at recovery | 1.014 | 0.992-1.037 | 0.203 |
|  | Number of BPA sessions | 1.554 | 1.030-2.345 | **0.036** |
|  | PAWP | 0.862 | 0.740-1.006 | 0.059 |
|  | Age | 1.009 | 0.963-1.057 | 0.708 |
| 3 | HR at recovery | 1.011 | 0.988-1.034 | 0.352 |
|  | Number of BPA sessions | 1.625 | 1.057-2.497 | **0.027** |
|  | PAWP | 0.842 | 0.715-0.991 | **0.038** |
|  | Age | 1.010 | 0.963-1.058 | 0.691 |
|  | NT-proBNP | 1.000 | 0.999-1.000 | 0.089 |
| 4 | HR at recovery | 1.010 | 0.987-1.034 | 0.380 |
|  | Number of BPA sessions | 1.626 | 1.058-2.499 | **0.027** |
|  | PAWP | 0.841 | 0.714-0.991 | **0.039** |
|  | Age | 1.010 | 0.964-1.059 | 0.674 |
|  | NT-proBNP | 1.000 | 0.999-1.000 | 0.179 |
|  | WHO FC | 0.800 | 0.253-2.532 | 0.704 |
| 5 | HR at recovery | 1.010 | 0.987-1.034 | 0.407 |
|  | Number of BPA sessions | 1.613 | 1.045-2.490 | **0.031** |
|  | PAWP | 0.838 | 0.710-0.990 | **0.038** |
|  | Age | 1.010 | 0.964-1.059 | 0.670 |
|  | NT-proBNP | 1.000 | 0.999-1.000 | 0.171 |
|  | WHO FC | 0.765 | 0.230-2.553 | 0.664 |
|  | mPAP | 1.006 | 0.959-1.055 | 0.805 |
| 6 | HR at recovery | 1.010 | 0.987-1.034 | 0.390 |
|  | Number of BPA sessions | 1.604 | 1.034-2.488 | **0.035** |
|  | PAWP | 0.849 | 0.719-1.003 | 0.054 |
|  | Age | 1.009 | 0.962-1.058 | 0.717 |
|  | NT-proBNP | 1.000 | 0.999-1.000 | 0.098 |
|  | WHO FC | 0.627 | 0.182-2.159 | 0.460 |
|  | PVR | 1.092 | 0.931-1.280 | 0.280 |
| 7 | ΔHR ^b^ | 1.019 | 0.994-1.044 | 0.138 |
|  | Number of BPA sessions | 1.553 | 1.030-2.343 | **0.036** |
|  | PAWP | 0.848 | 0.725-0.991 | **0.039** |
| 8 | ΔHR | 1.022 | 0.994-1.051 | 0.119 |
|  | Number of BPA sessions | 1.568 | 1.037-2.371 | **0.033** |
|  | PAWP | 0.849 | 0.725-0.994 | **0.042** |
|  | Age | 1.013 | 0.966-1.062 | 0.602 |
| 9 | ΔHR | 1.018 | 0.989-1.047 | 0.221 |
|  | Number of BPA sessions | 1.631 | 1.061-2.506 | **0.026** |
|  | PAWP | 0.831 | 0.705-0.981 | **0.029** |
|  | Age | 1.013 | 0.966-1.063 | 0.590 |
|  | NT-proBNP | 1.000 | 0.999-1.000 | 0.098 |
| 10 | ΔHR | 1.017 | 0.988-1.047 | 0.249 |
|  | Number of BPA sessions | 1.632 | 1.062-2.507 | **0.025** |
|  | PAWP | 0.831 | 0.704-0.982 | **0.029** |
|  | Age | 1.013 | 0.966-1.063 | 0.585 |
|  | NT-proBNP | 1.000 | 0.999-1.000 | 0.175 |
|  | WHO FC | 0.848 | 0.264-2.724 | 0.783 |
| 11 | ΔHR | 1.017 | 0.988-1.047 | 0.249 |
|  | Number of BPA sessions | 1.607 | 1.039-2.485 | **0.033** |
|  | PAWP | 0.827 | 0.698-0.979 | **0.028** |
|  | Age | 1.014 | 0.967-1.064 | 0.565 |
|  | NT-proBNP | 1.000 | 0.999-1.000 | 0.158 |
|  | WHO FC | 0.797 | 0.238-2.667 | 0.713 |
|  | mPAP | 1.009 | 0.963-1.058 | 0.695 |
| 12 | ΔHR | 1.019 | 0.990-1.050 | 0.206 |
|  | Number of BPA sessions | 1.589 | 1.022-2.471 | **0.040** |
|  | PAWP | 0.838 | 0.708-0.993 | **0.042** |
|  | Age | 1.014 | 0.966-1.064 | 0.588 |
|  | NT-proBNP | 1.000 | 0.999-1.000 | 0.086 |
|  | WHO FC | 0.660 | 0.191-2.284 | 0.512 |
|  | PVR | 1.104 | 0.941-1.296 | 0.224 |
| 13 | HR acceleration time ^c^ | 1.003 | 0.999-1.007 | 0.138 |
|  | Number of BPA sessions | 1.557 | 1.042-2.327 | **0.031** |
|  | PAWP | 0.843 | 0.719-0.989 | **0.036** |
| 14 | HR acceleration time | 1.003 | 0.999-1.007 | 0.138 |
|  | Number of BPA sessions | 1.563 | 1.042-2.344 | **0.031** |
|  | PAWP | 0.844 | 0.719-0.991 | **0.038** |
|  | Age | 1.003 | 0.960-1.048 | 0.892 |
| 15 | HR acceleration time | 1.002 | 0.998-1.006 | 0.240 |
|  | Number of BPA sessions | 1.624 | 1.065-2.475 | **0.024** |
|  | PAWP | 0.825 | 0.697-0.976 | **0.025** |
|  | Age | 1.006 | 0.962-1.051 | 0.801 |
|  | NT-proBNP | 1.000 | 0.999-1.000 | 0.093 |
| 16 | HR acceleration time | 1.002 | 0.998-1.006 | 0.271 |
|  | Number of BPA sessions | 1.626 | 1.066-2.480 | **0.024** |
|  | PAWP | 0.825 | 0.697-0.977 | **0.026** |
|  | Age | 1.006 | 0.963-1.052 | 0.784 |
|  | NT-proBNP | 1.000 | 0.999-1.000 | 0.166 |
|  | WHO FC | 0.855 | 0.266-2.746 | 0.793 |
| 17 | HR acceleration time | 1.002 | 0.998-1.006 | 0.265 |
|  | Number of BPA sessions | 1.600 | 1.043-2.454 | **0.031** |
|  | PAWP | 0.820 | 0.690-0.974 | **0.024** |
|  | Age | 1.007 | 0.963-1.053 | 0.756 |
|  | NT-proBNP | 1.000 | 0.999-1.000 | 0.147 |
|  | WHO FC | 0.798 | 0.239-2.670 | 0.714 |
|  | mPAP | 1.011 | 0.964-1.060 | 0.663 |
| 18 | HR acceleration time | 1.003 | 0.999-1.007 | 0.170 |
|  | Number of BPA sessions | 1.578 | 1.025-2.431 | **0.038** |
|  | PAWP | 0.831 | 0.700-0.988 | **0.036** |
|  | Age | 1.005 | 0.962-1.051 | 0.811 |
|  | NT-proBNP | 1.000 | 0.999-1.000 | 0.069 |
|  | WHO FC | 0.650 | 0.189-2.234 | 0.494 |
|  | PVR | 1.126 | 0.953-1.331 | 0.164 |

BPA, balloon pulmonary angioplasty; HR, heart rate; HRR1, heart-rate recovery at one minute; mPAP, Mean pulmonary arterial pressure; NT-proBNP, N-terminal pro-brain natriuretic peptide; PAWP, Pulmonary arterial wedge pressure; PVR, Pulmonary vascular resistance; WHO FC, World Health Organization functional class. ^a^ The value of HR at the moment when exercise stopped. ^b^ HR at peak minus HR at rest. ^c^ The time taken to increase to 75% of ΔHR (3min of rest was not included).
